# Identification and characterization of microRNAs in the intestinal tissues of sheep (*Ovis aries*)

**DOI:** 10.1371/journal.pone.0193371

**Published:** 2018-02-28

**Authors:** Lei Hou, Zhibin Ji, Guizhi Wang, Jin Wang, Tianle Chao, Jianmin Wang

**Affiliations:** Shandong Provincial Key Laboratory of Animal Biotechnology and Disease Control and Prevention, Shandong Agricultural University, Taian City, Shandong Province, China; Laboratoire de Biologie du Développement de Villefranche-sur-Mer, FRANCE

## Abstract

Sheep are small ruminants, and their long intestines exhibit high digestive and absorptive capacity in many different rearing conditions; however, the genetic bases of this characteristic remains unclear. MicroRNAs (miRNAs) play a major role in maintaining both intestinal morphological structure as well as in regulating the physiological functions of this organ. However, no study has reported on the miRNA expression profile in the intestinal tissues of sheep. Here, we analyzed and identified the miRNA expression profile of three different intestinal tissues (i.e., duodenum, cecum, and colon) of sheep (*Ovis aries*) using high-throughput sequencing and bioinformatic methods. In total, 106 known miRNAs were identified, 458 conserved miRNAs were detected, 192 unannotated novel miRNAs were predicted, and 195 differentially expressed miRNAs were found between the different tissues. Additionally, 3,437 candidate target genes were predicted, and 17 non-redundant significantly enriched GO terms were identified using enrichment analysis. A total of 99 candidate target genes were found to significantly enriched in 4 KEGG biological pathways. A combined regulatory network was constructed based on 92 metabolism-related candidate target genes and 65 differentially expressed miRNAs, among which 7 miRNAs were identified as hub miRNAs. Via these mechanisms, miRNAs may play a role in maintaining intestinal homeostasis and metabolism. This study helps to further explain the mechanisms that underlie differences in tissue morphology and function in three intestinal segments of sheep.

## Introduction

Ruminants have a unique digestive system compared with monogastric animals. Specifically, the gastrointestinal tract of ruminants harbors a large number of microflora that can transform and utilize dietary crude fiber [1]. Conventional research has primarily focused on the complex forestomach of ruminants, while there are few studies on the function of the small and large intestines [2]. Sheep are small ruminants whose small intestine is long and thin, being 27-fold longer than the body of the animal. The volume of the small intestine constitutes 22% of the total volume of the digestive tract and contains the most digestive enzymes in sheep. The duodenum is the most proximal segment and is characterized by the densest villi, the largest diameter, and the deepest position in the small intestine. The duodenum receives pancreatic juice from the pancreas and bile from the liver, aiding the intestines in the digestion of starch, fat, and protein. Following the rumen, the cecum and proximal colon of ruminants are the second largest sites of fermentation. Large amounts of cell wall carbohydrates, cellulose, and hemicellulose enter the cecum and colon, wherein they are fermented and utilized. The produced volatile fatty acids are rapidly taken up by the cecum and colon. The energy conversion efficiency of these two organs has been reported to be higher compared with that of the rumen [3, 4, 5]. The intestines of ruminants perform unique physiological functions, indicating that molecular regulation in the intestines is far more complicated than previously thought.

In recent years, the functions of miRNAs in the intestines have been extensively explored, with many novel miRNAs being identified. MiRNAs have been found to participate widely in the maintenance of intestinal homeostasis and the regulation of metabolic homeostasis [6, 7]. Moreover, miRNAs play an important regulatory role in intestinal mucosal immunity, stress resistance, the development and maintenance of intestinal morphological structure, as well as the regulation of various intestinal functions [7–10]. Studies have found that miRNAs are widely expressed in the intestinal tissues of pigs [11–13], mice [14, 15], and cattle [16, 17]. Some miRNAs are only found in particular intestinal segments, suggesting that miRNAs may play important roles in particular intestinal segments. The miRBase21.0 database lists a total of 793 bovine miRNAs, while only 153 miRNAs are identified in sheep. There have been previous studies of miRNAs in different sheep tissues, such as muscle [18], ovary [19], fat [20,21], wool follicle [22], and heart [23]. Currently, there is a lack of research regarding the miRNAs expression profile in the duodenum, cecum, and colon of sheep. Thorough and in-depth studies of the regulation of miRNAs expression in the intestines of sheep have vital scientific implications for revealing the regulatory mechanisms that affect the intestinal digestion and metabolism in sheep.

In the present study, we compared the miRNAs expression profile between the duodenum, cecum, and colon of sheep (*Ovis aries*) using Illumina-Solexa high-throughput sequencing technology. These results provide a reference for understanding the regulatory mechanism of digestion, absorption, and metabolism in different intestinal regions of sheep and for the generation of high-yield, high-efficiency breeds.

## Materials and methods

### Ethics statement of experimental animals

All of the animal experiments in this study were approved by the Ethics Committee of Animal Protection and Use at Shandong Agricultural University (License No.: 2004006). The experiments were performed in accordance with the “Experimental Animal Guidelines” put forth by the Ministry of Science and Technology (Beijing, China). All of the surgical procedures followed the recommendations proposed by the European Commission in 1997. Animals used in this study were the same as those used in our previous study [24], and were slaughtered in the same way.

### Sample collection and total RNA extraction

The experimental animals were three 11-month-old purebred ewes (*Ovis aries*) of similar weight. The animals were obtained from the same farm (Linqv Huanong Sheep Farm, Weifang, Shandong, China) and reared in consistent environments, i.e., the sheep were fed in stables, in natural light and without temperature control, free access to water and food.

The animals were slaughtered quickly to collect approximately intestinal tissues. All animals used in this study were handled in strict accordance with good clinical practices and all efforts were made to minimize suffering. Intestinal tissues were taken from the intestinal epithelium of the duodenum, the cecum, and the proximal colon immediately after slaughter. The samples were preserved in liquid nitrogen until use. Total RNA was extracted from the intestinal tissues using TRIzol. The integrity and concentration of the extracted RNA were determined using an Agilent 2100 Bioanalyzer. Samples with a RIN score >7.5 were used for library construction.

### Library construction and sequencing

The libraries were constructed following the mRNA preparation procedures for Illumina sequencer samples, with appropriate modifications. Specifically, total RNA from the duodenum, cecum and colon of three sheep was separately pooled, Illumina TruSeq samll RNA Sample Prep Kit (Cat#RS-200-0012) was used with 1 ug of total RNA for the construction of sequencing libraries. three libraries were constructed (Duodenum, DU; Cecum, CE; and Colon, CO), which were prepared for sequencing using standard Illumina protocols. All of the sequencing was performed in the Beijing Genomics Institute (BGI, Shenzhen, China). Sequencing data had been submitted to GEO database (GSE100649).

### Sequence data analysis and miRNAs identification

Raw sequencing image data were converted to raw reads via base calling. The following reads were removed: low-quality reads, reads with contaminated 5'-adapters, reads lacking 3'-adapters, reads lacking an insertion sequence, reads containing polyA sequences, and small fragments (<18 nt). A set of clean reads was ultimately obtained for analysis. Generally, the whole workflow is shown in S1 Fig.

To analyze sequence distribution and expression, the clean reads were mapped to the genome of *Ovis aries* (assembly Oar_v4.0, ftp://ftp.ncbi.nlm.nih.gov/genomes/all/GCF/000/298/735/GCF_000298735.2_Oar_v4.0/GCF_000298735.2_Oar_v4.0_genomic.fna.gz) using SOAP aligner v2.21 [25]. To identify the known miRNAs, the clean reads were compared with known mammalian precursor and mature miRNAs in the miRBase21.0 database [26] using BLAST. The criteria were as follows: Clean reads can be completely aligned to precursor (no mismatch), then compared with the mature miRNA, which allow dislocation, but at least there were 16 base coverage without mismatch. Those miRNAs whose reads number less than 10 in each sample were removed as false positive. The clean reads were compared with the Genbank (ftp://ftp.ncbi.nlm.nih.gov/genbank/) and Rfam v11.0 (http://rfam.janelia.org/) [27] databases to remove rRNA, scRNA, snoRNA, snRNA, and tRNA sequences. The clean reads that were not matched in the databases but were matched to the antisense strand of exons, introns, and intergenic regions in the genome were used to predict novel miRNAs with miReap (http://mireap.sourceforge.net/). Using miReap, we predicted novel miRNA precursor hairpins with a minimum free energy ≤ 18 (kcal/mol), while all other parameters were settled as default. Then, retained clean reads were compared with predicted novel miRNA precursors using a same method and parameters of our known miRNA identification, and all successfully mapped reads were automatic constructed into novel miRNAs.

For the comparison analysis of novel miRNAs, we collected 2,381 sheep unannotated novel miRNAs from other 8 sheep miRNA sequencing studies [20, 23, 28–33]. With at least 18 base coverage and not more than one mismatch, and e-value < 1e-5, the comparison analysis of novel miRNAs was performed with BLAST.

### Differential expression of miRNAs and prediction of target genes

The expression levels of known and novel miRNAs were normalized to the same order of magnitude to get the expression of transcript per million (TPM).

Normalization formula: Normalized expression = Actual miRNA count/Total count of clean reads*1000000.

The standardized results were then used to calculate fold-change and P-value to analyze the fold differences between different samples.

Fold-change formula: Fold_change = log2(treatment/control)

*P*-value formula:

$$\begin{array}{c} p(y|x)={\left(\frac{N_{2}}{N_{1}}\right)}^{y}\frac{(x+y)!}{x!y!{\left(1+\frac{N_{2}}{N_{1}}\right)}^{\left(x+y+1\right)}}\\ C(y\leq y_{\min}|x)=\sum_{y=0}^{y\leq y_{\min}}p(y|x)\;D(y\geq y_{\max}|x)=\sum_{y=y_{\max}}^{\infty }p(y|x) \end{array}$$

The *P*-value was calculated based on Poisson distribution to determine the significance of any difference in miRNA expression between different samples. And the n, the Bonferroni corrected P-value was calculated. The significantly differentially expressed miRNAs were declared at a fold change ≥ 2 and a Bonferroni corrected P-value <0.01.

Differentially expressed miRNAs were identified and their predicted target genes were predicted using miRanda [34], and TargetScan v7.0 [35] software. Gene sequences used for target prediction analysis were downloaded from (ftp://ftp.ncbi.nlm.nih.gov/genomes/all/GCF/000/298/735/GCF_000298735.2_Oar_v4.0/GCF_000298735.2_Oar_v4.0_rna.fna.gz). For the TargetScan v7.0, a seed match ≥ 7 and context+ score percentile ≥ 99 was settled as the threshold. And for the miRanda, a free energy ≤ -20 (kcal/mol) was chosen as a cutoff. The intersection of these prediction results was taken as the set of candidate target genes.

### GO annotation and KEGG pathway analysis

To generate a comprehensive description of the biological characteristics of candidate target genes, we mapped all of the candidate target genes to the Gene Ontology (GO) [36] database using DAVID v6.8 online software with the FDR <0.1, and background of genes with FPKM>1 from previous report [37], other parameters are in accordance with the DAVID default settings [38]. The GO terms were subjected to classification and enrichment analysis regarding molecular function, cellular component, and biological process. Significantly enriched GO terms were calculated using the hypergeometric test with all genes in the reference genome as the background. In addition, we mapped all of the candidate target genes to the Kyoto Encyclopedia of Genes and Genomes (KEGG, http://www.genome.jp/kegg/) [39] database to obtain significantly enriched metabolism and signal transduction pathways. Significant enrichment was identified at FDR <0.1.

### Regulatory network construction

The interactions between filtered candidate target genes (filter criteria: combined score ≥0.4) were explored using the STRING 10.0 databas [40]. An interactive regulatory network between miRNAs and candidate target genes was generated using Cytoscape v3.4 [41].

### Real-time quantitative RT-PCR validation

We randomly selected 14 differentially expressed miRNAs (4 known miRNAs and 10 novel miRNAs) for quantitative validation by real-time quantitative PCR. The primers were designed with mature miRNA sequences as the templates. Base modification was performed at the 5'-end if necessary (S1 Table). U6 was chosen as the reference gene.

Each miRNA was tested using three biological replicates, and each biological replicate had three technical replicates. The expression of each miRNA was calculated based on a standard curve using the 2^-ΔΔCT^ method, and the expression is presented as 2^-ΔΔCT^ ± SEM. The significance of difference in miRNA expression between three intestinal segments was tested using one-way ANOVA (P ≤0.05).

## Results

### Sequence data from three segments of intestinal tissues

**Fig 1:**
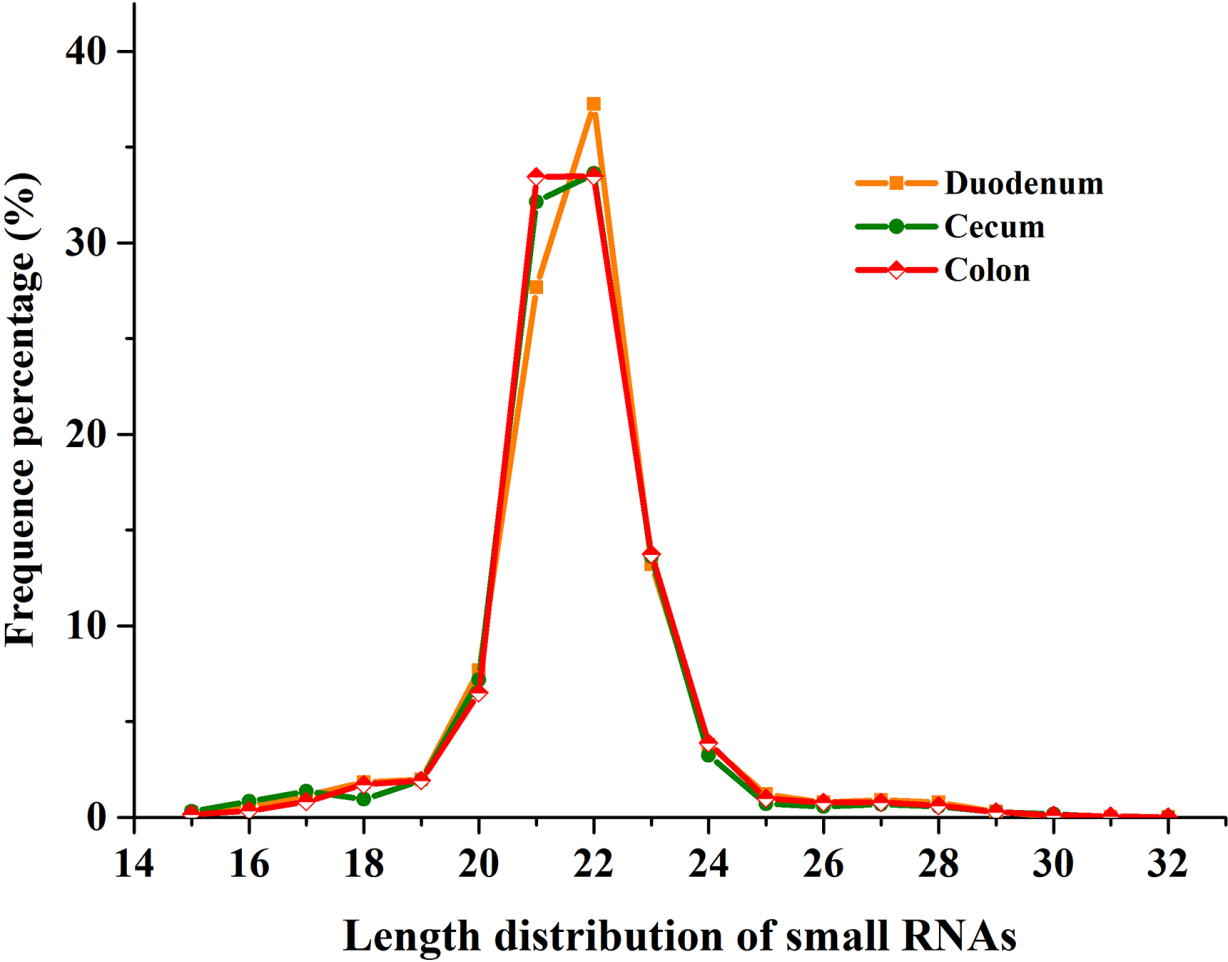
Length distribution of small RNAs in three libraries. Sequence length distribution of clean reads based on the abundance and distinct sequences; the most abundant size class was 22 nt, followed by 21 nt, 23 nt and 20nt.

In the DU, CE, and CO libraries, we obtained 28,493,897; 27,836,933, and 26,057,470 high-quality reads, respectively. After removal of reads lacking 3'-adapters, reads with 5'-contamination, reads <18 nt, reads lacking insertion sequences, and reads containing polyA sequences, we obtained 27,498,891, 26,490,229, and 25,351,117 clean reads in the three libraries (96.51%, 95.16%, and 97.29% of the high quality reads, respectively, Table 1). The length of sRNAs obtained by sequencing generally ranged from 19 to 24 nt, accounting for 91.62%, 91.76%, and 93% of the total reads in the DU, CE, and CO libraries, respectively. Among these reads, those of 22 nt were the most abundant, constituting 37.26%, 33.62%, and 33.48% of the DU, CE, and CO libraries, respectively. Reads of 21 and 23 nt in length were the next most abundant. The distribution of read length was in agreement with the length distribution pattern of miRNA sequences in mammals (Fig 1).

**Table 1:** Distribution of total sequence for small RNAs in small-tailed han sheep.

Category	Duodenum		Cecum		Colon	
	Total	Percent	Total	Percent	Total	Percent
**Raw reads**	28,536,048	N/A	27,878,234	N/A	26,097,405	N/A
**High quality reads**	28,493,897	100%	27,836,933	100%	26,057,470	100%
**3’ adapter null**	268,000	0.94%	443,102	1.59%	248,668	0.95%
**Insert null**	19,580	0.07%	443,102	1.59%	15,055	0.06%
**5’ adapter contaminants**	9,650	0.03%	9,981	0.04%	8,662	0.03%
**Smaller than 18 nt**	697,631	2.45%	874,414	3.14%	433,880	1.67%
**PolyA**	145	0.00%	135	0.00%	88	0.00%
**Clean reads**	27,498,891	96.51%	26,490,229	95.16%	25,351,117	97.29%
**Mapping on sheep genome**	16,733,785	60.85%	17,005,544	64.20%	16,567,397	65.35%

In the DU, CE, and CO libraries, the total numbers of clean reads mapped to the genome were 16,733,785, 17,005,544, and 16,567,397, respectively (60.85%, 64.20%, and 65.35% of the total reads, respectively, Table 1). Chromosomes Chr5, Chr2, and Chr11 harbored the largest number of reads, whereas Chr25 and Chr26 harbored the smallest number of reads (S2 Table).

**Fig 2:**
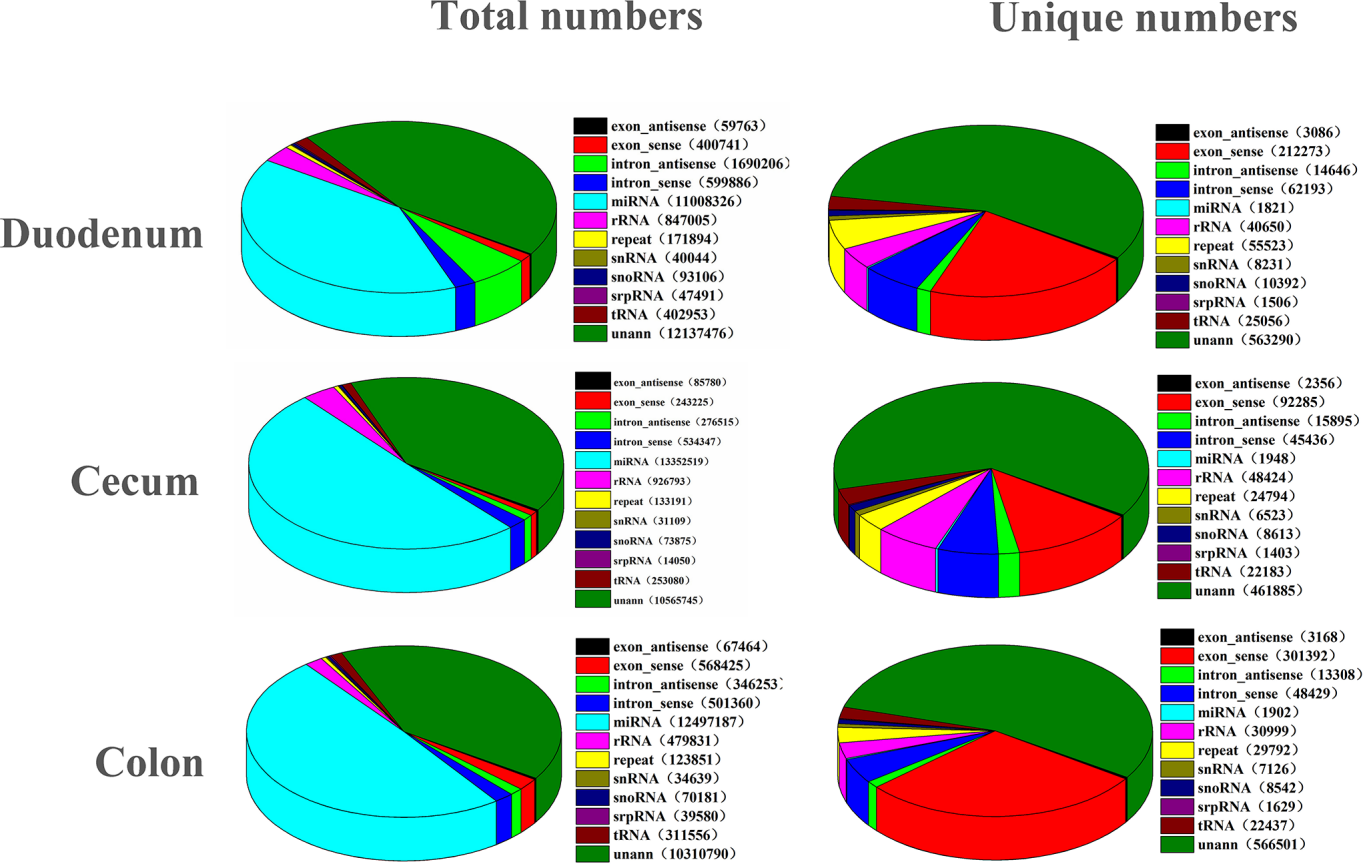
Classification of small RNAs in three libraries. The clean reads were annotated and classified as miRNA, rRNA, tRNA, snRNA and snoRNA in GenBank and Rfam databases, and partial reads were not annotated.

To obtain the components contained in clean reads, we classified and annotated the sRNAs. In the DU, CE, and CO libraries, a total of 13,432,974, 14,651,426, and 12,438,925 reads, respectively, were annotated as various types of RNAs (rRNAs, tRNAs, snRNAs, snoRNAs, and miRNAs). Annotated repeat reads represented 123,851, 133,191, and 171,894 reads in the DU, CE, and CO libraries, respectively. Moreover, there were 10,310,790, 10,565,745, and 12,137,476 unannotated reads in the three DU, CE, and CO libraries. These unannotated reads were used in the subsequent prediction of novel miRNAs (Fig 2).

### The identification of known miRNAs and novel miRNAs

To identify known miRNAs, we aligned the clean reads against 106 precursor miRNAs and 153 mature miRNAs of sheep in the miRBase 21.0 database. A total of 106 mature miRNAs were detected in the three libraries (S5 Table). These mature miRNAs were derived from 92 precursor miRNAs and distributed in 51 miRNA families. Of the clean reads, 40%, 50.4%, and 49.3% were matched to precursor miRNAs. But these miRNAs only accounted for 0.18%, 0.27%, and 0.18% of total unique sRNAs in the DU, CE, and CO libraries, respectively. In the DU, CE, and CO libraries, miR-143 had the largest number of reads, while the miR-21, miR-148a, miR-26, and let-7 families were the next most abundant.

The sRNAs that were unannotated to sequences of known miRNAs in the miRBase21.0 database but mapped to the antisense strand of exons, introns, and intergenic regions were selected for predicting novel miRNAs using miReap. The reads with predicted hairpin structures were mapped to known mammalian precursor and mature miRNAs in the miRBase21.0 database, and the remaining unmapped but hairpin-structured reads were considered potential new miRNAs without known miRNA annotations. In total, 458 miRNAs were conserved with other species, 192 miRNAs were identified as unannotated novel miRNAs in the three libraries (S5 Table). Moreover, to get further information on new miRNAs, we collected sheep unannotated novel miRNAs from other studies [20, 23, 28–33], and a comparison analysis has been performed. As a result, 71 novel miRNAs obtained acceptable results with other studies (S6 Table).

### Analysis of differentially expressed miRNAs and prediction of target genes

A total of 195 known, conserved and novel differentially expressed miRNAs were identified in the DU, CE, and CO libraries (S7 Table). There were 131 known and novel differentially expressed miRNAs between the DU and CE libraries, with 81 being expressed at higher levels in the DU library and 50 being expressed more highly in the CE library. 123 known and novel differentially expressed miRNAs between the CO and DU libraries, with 49 being expressed more highly in the CO library and 74 being expressed at higher levels in the DU library. Lastly, there were 61 known and novel miRNAs that were differentially expressed between the CO and CE libraries, with 29 being expressed at higher levels in the CO library and 32 being expressed more highly in the CE library.

The 195 known, conserved and novel differentially expressed miRNAs were used to predict target genes using miRanda, and Targetscan. A total of 3,437 non-redundant high-confidence candidate target genes were obtained from the intersection of the obtained gene sets (S8 Table). Newly predicted novel_miR_258 had the largest number of predicted target genes, at 178. There were 9 miRNAs with over 100 predicted target genes, and 97 miRNAs with 10–100 predicted target genes, while 74 miRNAs with less than 10 predicted target genes. *LOC101109384* and *USP54* were the candidate target genes with the largest number of miRNAs (8 miRNAs), followed by *DPF3*, *GFM2*, *MAP2*, and *DOCK5* (6 miRNAs).

**Fig 3:**
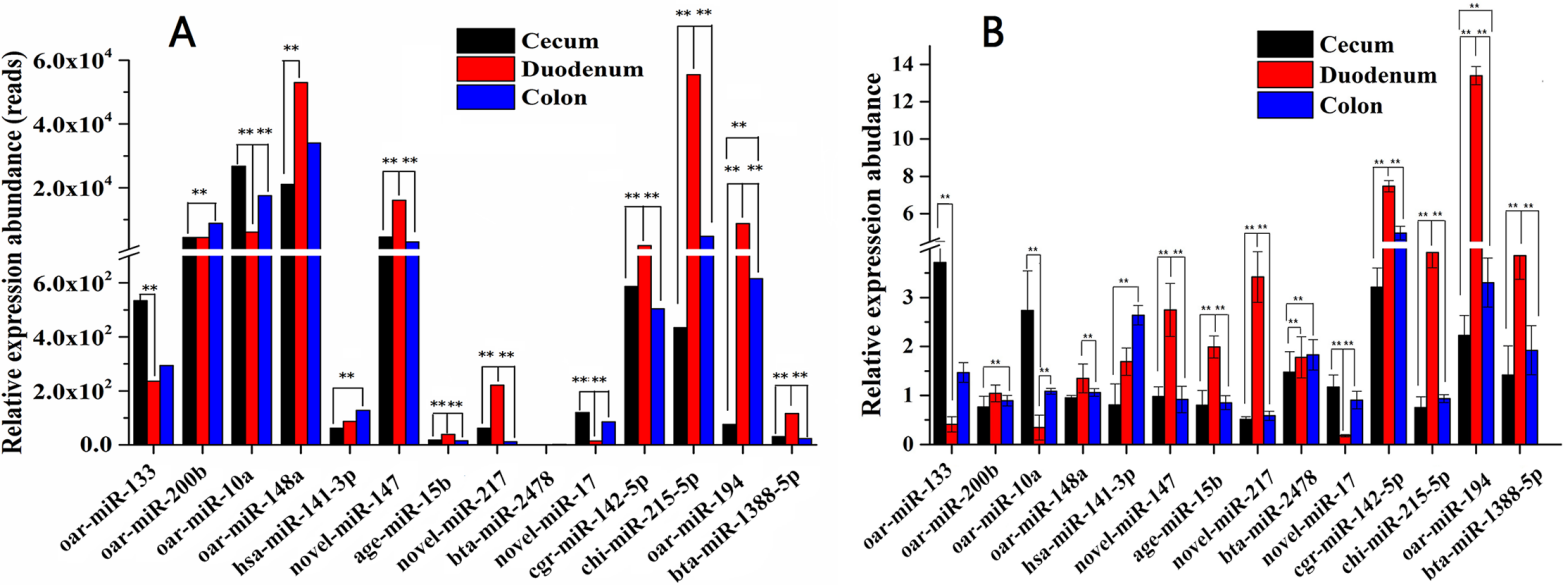
Validation of differentially expressed miRNAs by qRT-PCR. A. Result of RNAseq. B. Result of qRT-PCR. oar-miR-10a, oar-miR-148a, oar-miR-200b oar-miR-133 and oar-miR-194 were known sheep miRNAs, cgr-miR-142-5p, chi-miR-215-5p, bta-miR-1388-5p, age-miR-15b, bta-miR-2478, and hsa-miR-141-3p were conserved miRNAs, novel_miR_17, novel_miR_147 and novel_miR_217 were novel miRNA candidates. The relative quantification of expression was calculated using the 2^-△△CT^ method after the threshold cycle (Ct) and was normalized with the Ct of U6. The relative expression levels were presented as the 2^-△△CT^ means ± SE. The error bars indicate the standard error of the 2^-△△CT^ mean values. * represents *p* <0.05, ** represents *p* <0.01.

The results of qRT-PCR validation for differentially expressed miRNAs showed that the expression trends of 12 miRNAs were consistent with the sequencing results. Only two miRNAs (oar-miR-200b and bta-miR-2478) showed different trends between the qPCR and sequencing data (Fig 3). Specifically, the sequencing results showed high-level expression of oar-miR-200b in the colon, whereas the quantitative PCR results showed relatively high expression levels of this miRNA in the duodenum. Meanwhile, the sequencing results showed the highest expression level of bta-miR-2478 in the cecum, whereas the qPCR results showed relatively high expression levels of this miRNA in the duodenum.

### GO enrichment for candidate target genes of differentially expressed miRNAs

To better illustrate the function of differentially expressed miRNAs and reveal their associated biological functions in different intestinal segments, we conducted GO enrichment analysis for 3,437 candidate target genes.

The 2,709 candidate target genes that corresponded to the differentially expressed miRNAs between the DU and CE samples were significantly enriched in 16 GO terms (FDR < 0.1). Specifically, 126 candidate target genes were significantly enriched in 2 biological processes, primarily “negative regulation of transcription from RNA polymerase II promoter” and “positive regulation of transcription from RNA polymerase II promoter”; 1,020 candidate target genes were significantly enriched in 8 cellular component terms, with most in “exosome” and “cytoplasm”; and 469 candidate target genes were significantly enriched in 6 molecular function terms, primarily “ATP binding” and “poly(A) RNA binding”.

The 2,443 candidate target genes corresponding to miRNAs that were differentially expressed between the DU and CO libraries were significantly enriched in 15 GO terms (FDR < 0.1). Specifically, 113 candidate target genes were significantly enriched in 2 biological processes, primarily “negative regulation of transcription from RNA polymerase II promoter” and “positive regulation of transcription from RNA polymerase II promoter”, 932 genes candidate target genes were significantly enriched in 8 cellular component terms, mostly “cytoplasm” and “extracellular exosome”; 408 candidate target genes were significantly annotated to 5 molecular function terms, with most in “ATP binding” and “poly(A) RNA binding”.

**Fig 4:**
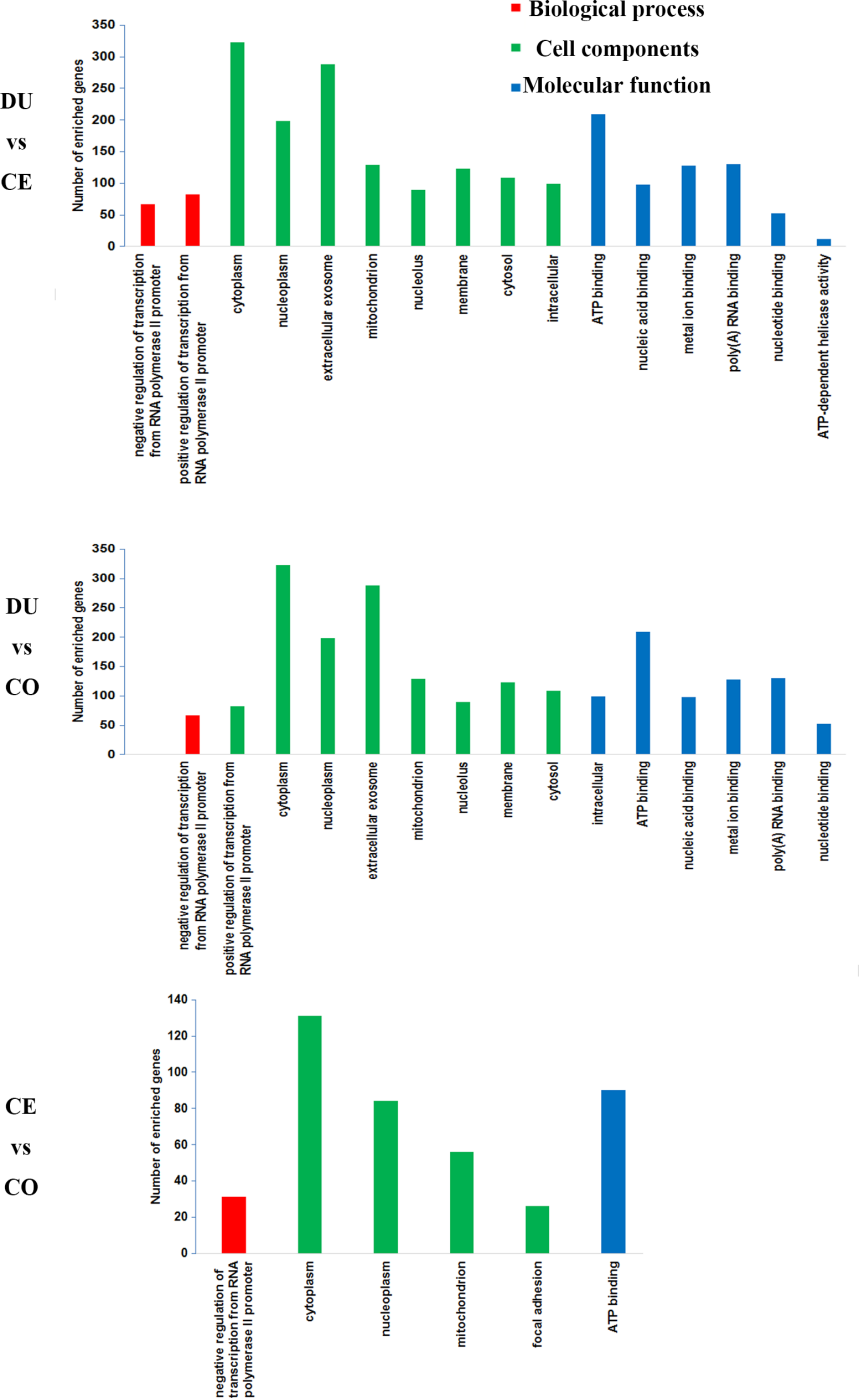
GO classification of 3,437 candidate target genes for 195 differentially expressed miRNAs. The figure shows GO enrichment for the candidate target genes in molecular function, cellular component and biological processes.

The 1,076 candidate target genes that were targeted by differentially expressed miRNAs between the CE and CO libraries were significantly enriched in 6 GO terms (FDR < 0.1). Specifically, 257 candidate target genes were significantly enriched in 4 cell components, primarily “cytoplasm” and “nucleoplasm”; 31 candidate target genes were significantly enriched in 1 biological processes, “negative regulation of transcription from RNA polymerase II promoter”; and 90 candidate target genes were significantly enriched in 1 molecular function term, “ATP binding”. A subset of the GO annotation results are provided in Fig 4 and S9 Table.

### KEGG analysis and regulatory network construction

To further understand the biological pathways that may be regulated by miRNAs, we performed KEGG pathway enrichment analysis using all 3,437 candidate target genes. KEGG pathway analysis showed that a total of 99 candidate target genes (DU vs CE, 44 genes; DU vs CO, 33 genes; CE vs CO, 29 genes) were significantly annotated to 4 pathways (DU vs CE, “MAPK signaling pathway”; DU vs CO, “Bacterial invasion of epithelial cells” and “Pancreatic cancer”; CE vs CO, “PI3K-Akt signaling pathway”). (S10 Table).

**Fig 5:**
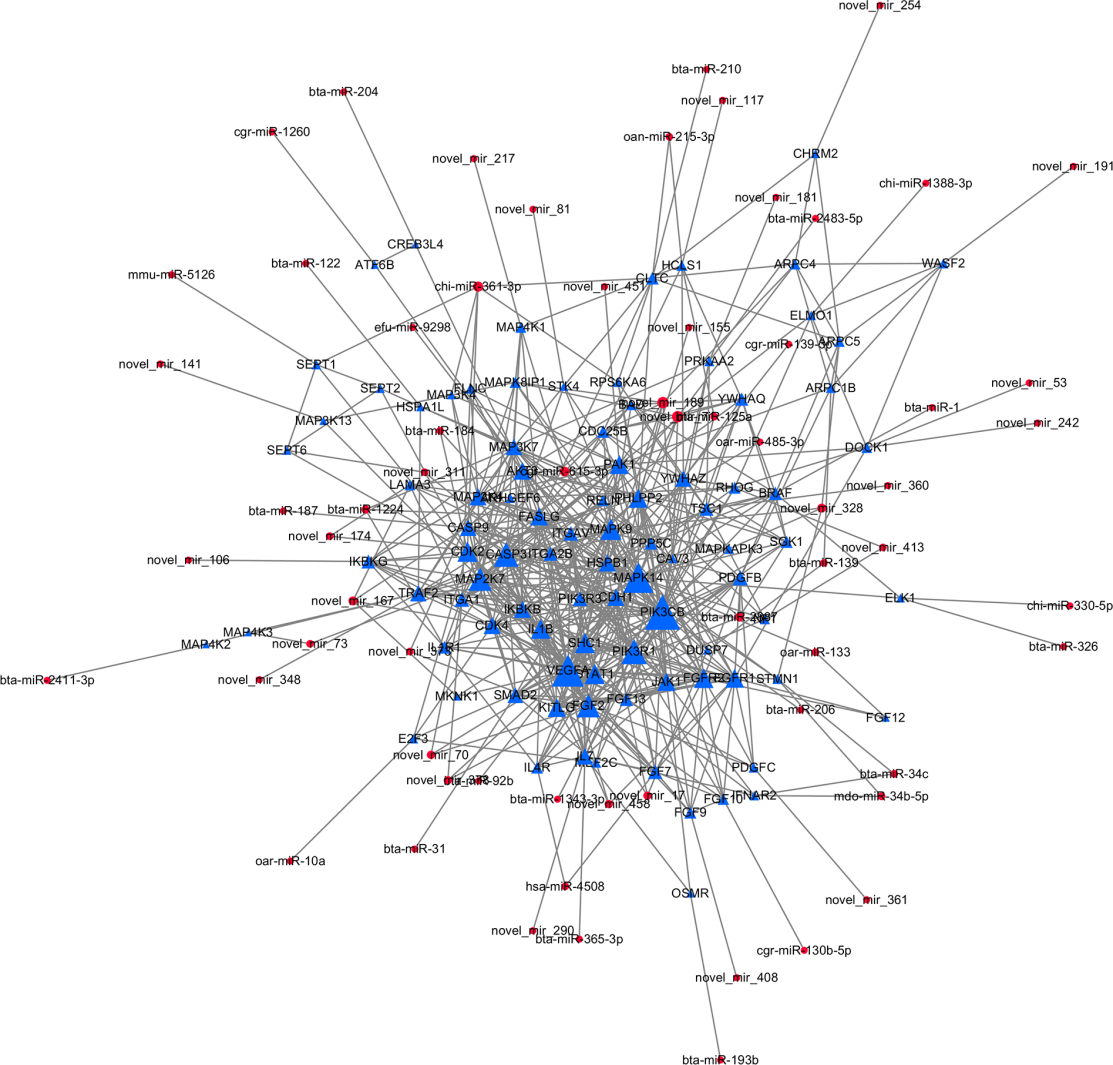
Regulatory network linking the differential expressed miRNAs and their predicted target genes. The node size was decided on the basis of the interaction degree value. Blue triangle node: Protein coding gene. Red circular node: miRNA.

In this study, we used the significantly enriched pathways as the base point to reveal the network regulatory relationship between miRNAs and their target genes. Using protein–protein interaction analysis in the STRING database, we identified 92 candidate target genes with interactions, which were regulated by 65 differentially expressed miRNAs. We cross-referenced these targets with the protein–protein interaction network using Cytoscape 3.4 and constructed a regulatory network of 157 nodes and 615 interactions (Fig 5). In this network, five genes were identified as hub genes (top 5% interaction degree), PIK3CB, VEGFA, MAPK14, PIK3R1, and CASP3, the 5 hub genes may perform important functions in the constructed regulatory network. Therefore, their corresponding predicted regulatory miRNAs were identified as hub miRNAs (PIK3CB: bta-miR-125a and bta-miR-206; VEGFA: novel-miR-70 and novel-miR-378; MAPK14: oar-miR-133; PIK3R1: bta-miR-1343-3p; CASP3: novel_mir_174).

## Discussion

miRNAs are a major class of post-transcriptional regulatory factors that have been heavily studied in recent years. miRNAs are extensively involved in various biological processes, such as development and metabolism. Despite extensive research on miRNAs in intestinal diseases and homeostasis, little work has been dedicated to the intestines of sheep, which exhibit a unique digestive system. Only one recent study examined miRNAs in sheep and investigated the miRNA expression profile of sheep intestines that were infected with parasites [42]. In the present study, based on sequencing of miRNA libraries from the duodenum, cecum, and colon of *Ovis aries*, we found that miRNAs were primarily concentrated on Chr18, while Chr5 had the highest distribution frequency of reads (S5 Table). Different chromosomes or chromosomal regions can be enriched in genes or other regulatory regions that govern certain functions. Analyzing the distribution of miRNAs on different chromosomes has great implications for investigating the regulatory functions of miRNAs. In the present study, only three known sheep miRNAs were detected on Chr5, which exhibited the highest distribution frequency of reads and accounted for more than 41.9% of the total reads. Our result indicates that there is indeed a bias in terms of the chromosomal distribution of differentially expressed miRNAs.

A typical feature of miRNAs is their strict spatiotemporal expression pattern. In this study, we identified 998,667, 731,745, and 1,035,225 unique sRNAs in the DU, CE, and CO libraries, respectively. There were large differences in the types of miRNAs between the different libraries. This result suggests that different miRNAs participate in the regulation of physiological functions of different intestinal segments by regulating their target genes, thereby mediating the unique physiological functions of these tissues. In the three libraries, miR-143 showed the highest expression level, with 4,606,500, 7,297,109, and 6,614,344 reads in the DU, CE, and CO libraries. According to previous research, miR-143 primarily performs important functions as a tumor suppressor. In the intestines, miR-143 primarily functions in colorectal cancer and post-cancer repair of intestinal epithelial injury [43] and is likely involved in nutrient digestion and absorption. Other highly expressed miRNAs, e.g., miR-10a and miR-10b, were upregulated in the CE and CO libraries. A previous study reported that miR-10a and miR-10b target Hox transcripts in several species and may play a major role in development [44]. Additionally, miR-10b is involved in cell cycle regulation and cancer recognition by the immune system [45, 46]. Taken together, the above information indicates that miR-10a and miR-10b may be related to the rapid regeneration of the intestinal mucosal epithelium in sheep. Moreover, bta-miR-215, hsa-miR-194-5p and bta-miR-192 were differentially expressed between the three types of intestinal tissues. The variation in miRNA expression levels between different intestinal segments may indicate the important regulatory roles of miRNAs in the development and normal maintenance of intestinal morphology and structure, as well as the performance of various tissue-specific functions.

Studies on miRNAs and their target genes are important for understanding the mechanisms of miRNAs in various biological processes [47, 48]. A total of 4,422 candidate target genes were obtained in the present study. Based on the observed regulatory relationship between miRNAs and their target genes, one miRNA might regulate multiple target genes; in turn, a given target gene could be regulated by numerous miRNAs. This result is consistent with those of previous studies [49].

The GO annotation and KEGG pathway enrichment analysis for target genes can provide a comprehensive description of the attributes of genes and gene products in organisms, helping to understand the biological functions and associated signaling pathways of candidate target genes. In the GO annotation, DU vs CE and DU vs CO showed almost exactly the same enrichment results, while the GO analysis result of CE vs CO is much more different. Such results are consistent with functional differences between intestinal segments. In the KEGG analysis, only 4 pathways achieved significant enrichment level. Although the three differentially comparison groups obtained a completely different KEGG enrichment result, (DU vs CE: MAPK signaling pathway; DU vs CO: Bacterial invasion of epithelial cells, Pancreatic cancer; CE vs CO: PI3K-Akt signaling pathway), their related functions are highly similar. All of the four pathways are functionally related to cell proliferation and apoptosis, which indicates the differentially expressed miRNAs might affect cell proliferation and apoptosis of different intestinal segments via targeted regulation of related genes, resulting in different intestinal functions.

The regulation of gene expression is extremely complex. To explore the intricate regulatory relationships between differentially expressed miRNAs, candidate target genes, and gene products, we constructed an interactive regulatory network comprising 65 miRNAs and 92 candidate target genes by exploring gene interactions in the STRING database. In this combined regulatory network, gene PIK3CB, VEGFA, MAPK14, PIK3R1, and CASP3 were identified as hub genes, and their related miRNAs were detected as hub miRNAs. As a potential related miRNA of PIK3CB, miR-206 was reported as a regulatory miRNA on cell proliferation and apoptosis [50, 51], and was identified with PIK3CB showing sensitive to initial injury intensity induced by freeze damage [52]. Another potential related miRNA of PIK3CB, miR-125a, was reported as important regulation miRNA on metabolism [53] and cell development [54]. However, the targeting relationships between miR-125a, miR-206 and PIK3CB are still not reported and verified. Furthermore, in the present study, miR-125a and miR-206 showed opposite expression patterns, miR-125a showed higher expression in cecum and colon, while miR-206 showed highest expression in duodenum. For VEGFA, potential related miRNAs novel-miR-70 and novel-miR-378 were both novel identified miRNAs. Comparative analysis reveals that novel-miR-70 and novel-miR-378 showed homology with unannotated novel miRNA “chr5_15893_mature” [20] and “gi-417531957-ref-NC_019462.1-:15688451:15688536:+” [33], while their detailed functions kept unknown. For gene MAPK14, oar-miR-133 has been identified as the only related miRNA. miR-133 has been reported as an important regulatory factor on cell apoptosis [55] and conversion [56]. The predicted relationship between miR-133 and MAPK14 may indicates that mir-133 may affect the function of different intestine segments by regulating the expression of MAPK14, while more experiments will be required to confirm this assumption. For gene PIK3R1, the only related miRNA is miR-1343-3p, which showed highest expression in colon. Recent studies have shown that miR-1343-3p has tumor suppressor effect on multiple cancers [57, 58]. And for gene CASP3, a cecum specifically expressed novel-miR-174 has been identified as the only related miRNA. To our knowledge, there is currently no report about novel-miR-174, which indicates novel-miR-174 could be a tissue-specific miRNA. In summary, the hub miRNAs detected in this regulatory network may participate in intestinal epithelial morphogenesis, development, and growth, playing a role in maintaining intestinal homeostasis and metabolism. However, the targeting relationships between hub miRNAs and hub genes were not verified, and their specific mechanisms of functions are still unknown. Further researches will be performed to confirm the specific functions of these miRNAs.

## Conclusions

In this study, we successfully constructed three sRNA libraries (DU, CE, and CO) in *Ovis aries*. In total, 106 known miRNAs, 458 conserved miRNAs, 192 unannotated novel miRNAs, and 195 differentially expressed miRNAs were identified by high-throughput sequencing and bioinformatics analysis. Additionally, 3,437 candidate target genes were significantly annotated to 17 GO terms and enriched in 4 KEGG biological pathways, such as “MAPK signaling pathway”, “Bacterial invasion of epithelial cells”, “Pancreatic cancer” and “PI3K-Akt signaling pathway”. Lastly, a combined regulatory network containing 92 candidate target genes and 65 differentially expressed miRNAs was constructed, among which 7 miRNAs were identified as hub miRNAs. The results provide a reference for elucidating, at the post-transcriptional level, the regulatory mechanisms that differentiate the morphological structures and physiological functions of the duodenum, cecum, and colon in sheep.

## Supporting information

S1 Fig Workflow for the differential expression analysis and functional annotation of miRNAs. (TIF)

S2 Fig Overview of miRNA annotation, identification, differential expression analysis and functional annotation results. (TIF)

S3 Fig Log2 transformed read distribution box plots for the three libraries. (TIF)

S1 Table Primer sequences for qRT-PCR validation. (XLSX)

S2 Table Distribution of reads and sheep known miRNAs on chromosome. (XLSX)

S3 Table Mireap novel miRNA precursor hairpins prediction results. (XLSX)

S4 Table Mireap novel miRNA construction results. (XLSX)

S5 Table Known, conserved and novel miRNAs identified in three libraries. (XLSX)

S6 Table Comparison results of unannotated novel miRNAs. (XLSX)

S7 Table Differentially expressed miRNAs between different libraries. (XLSX)

S8 Table Targetscan and miRanda differentially expressed miRNAs target prediction results. (XLSX)

S9 Table GO functional enrichment for candidate target genes. (XLSX)

S10 Table KEGG pathway annotations for candidate target genes. (XLSX)
