# Supplementary figures and images for: Identification and characterization of microRNAs in the intestinal tissues of sheep (Ovis aries)
Source: PLoS One. 2018 Feb 28;13(2):e0193371. doi: 10.1371/journal.pone.0193371 (PMC5831392; doi:10.1371/journal.pone.0193371)

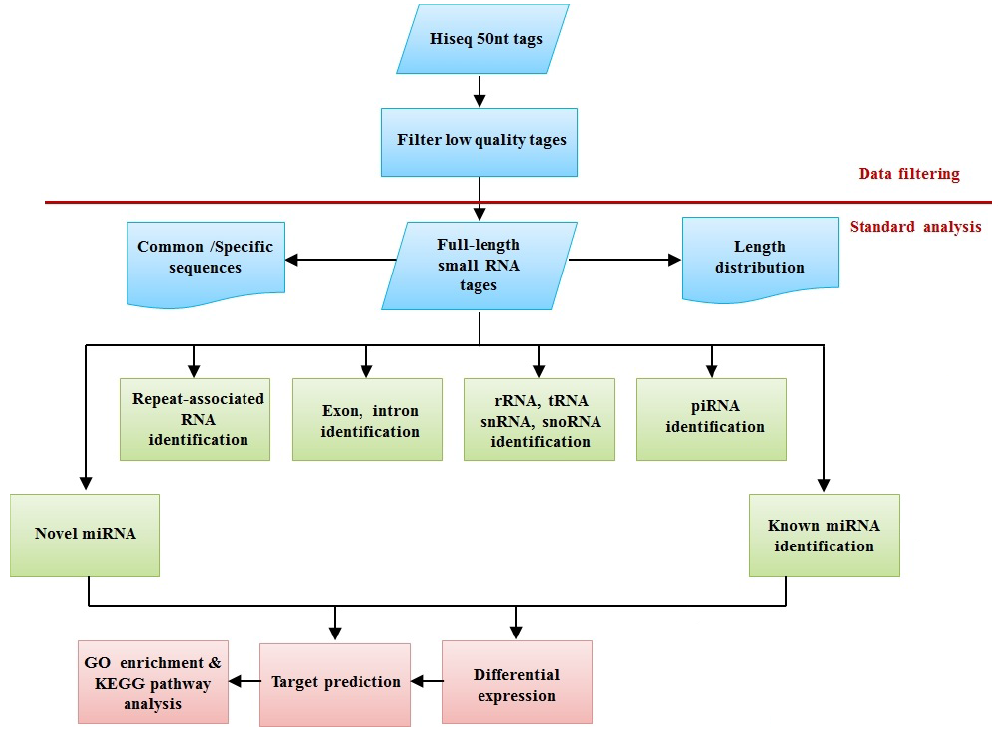

Supplement: S1 Fig — (TIF) [file pone.0193371.s001.tif]

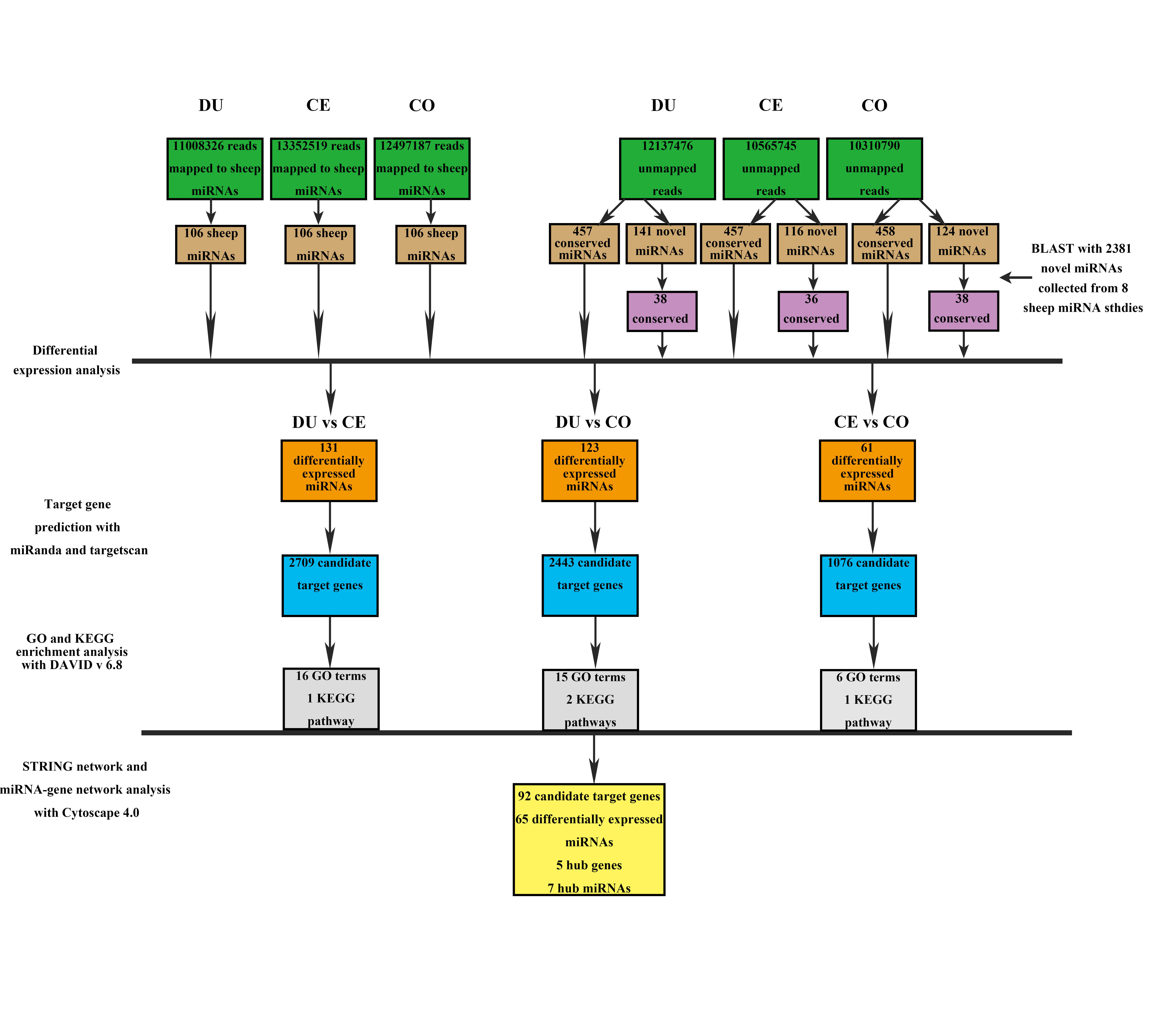

Supplement: S2 Fig — (TIF) [file pone.0193371.s002.tif]

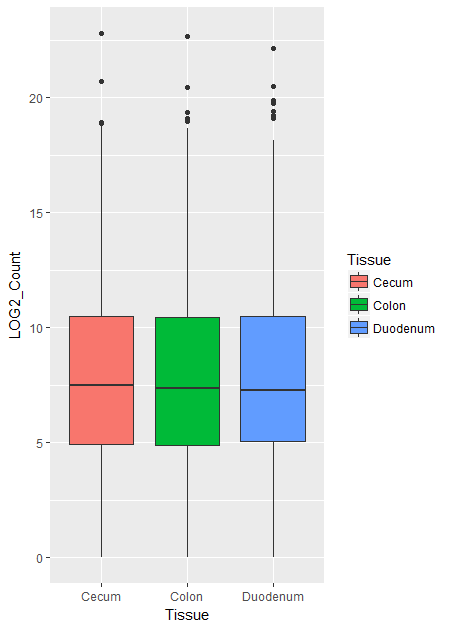

Supplement: S3 Fig — (TIF) [file pone.0193371.s003.tif]
